# Local excision vs. proctectomy in patients with ypT0–1 rectal cancer following neoadjuvant therapy: a propensity score matched analysis of the National Cancer Database

**DOI:** 10.1007/s10151-024-02994-4

**Published:** 2024-09-21

**Authors:** N. Horesh, S. H. Emile, M. R. Freund, Z. Garoufalia, R. Gefen, A. Nagarajan, S. D. Wexner

**Affiliations:** 1 Ellen Leifer Shulman and Steven Shulman Digestive Disease Center, Cleveland Clinic Florida, 2950 Cleveland Clinic Blvd., Weston, FL 33331 USA; 2 Department of Surgery and Transplantations, Sheba Medical Center, Ramat Gan, Israel; 3 Tel Aviv University, Tel Aviv, Israel; 4 Colorectal Surgery Unit, Faculty of Medicine, Mansoura University, Mansoura, Egypt; 5 Department of General Surgery Shaare Zedek Medical Center, Faculty of Medicine, Hebrew University of Jerusalem, Jerusalem, Israel; 6 Department of General Surgery, Hadassah Medical Organization and Faculty of Medicine, Hebrew University of Jerusalem, Jerusalem, Israel; 7 Department of Hematology/Oncology, Cleveland Clinic Florida, Weston, FL USA

**Keywords:** Neoadjuvant, Local excision, Proctectomy, Rectal Cancer, Survival

## Abstract

**Background:**

We aimed to evaluate outcomes of organ preservation by local excision (LE) compared to proctectomy following neoadjuvant therapy for rectal cancer.

**Methods:**

This retrospective observational study using the National Cancer Database (NCDB) included patients with locally advanced non-metastatic rectal cancer (ypT0–1 tumors) treated with neoadjuvant therapy between 2004 and 2019. Outcomes of patients who underwent LE or proctectomy were compared. 1:1 propensity score matching including patient demographics, clinical and therapeutic factors was used to minimize selection bias. Main outcome was overall survival (OS).

**Results:**

11,256 of 318,548 patients were included, 526 (4.6%) of whom underwent LE. After matching, mean 5-year OS was similar between the groups (54.1 vs. 54.2 months; *p* = 0.881). Positive resection margins (1.2% vs. 0.6%; *p* = 0.45), pathologic T stage (*p* = 0.07), 30-day mortality (0.6% vs. 0.6%; *p* = 1), and 90-day mortality (1.5% vs. 1.2%; *p* = 0.75) were comparable between the groups. Length of stay (1 vs. 6 days; *p* < 0.001) and 30-day readmission rate (5.3% vs. 10.3%; *p* = 0.02) were lower in LE patients. Multivariate analysis of predictors of OS demonstrated male sex (HR 1.38, 95% CI 1.08–1.77; *p* = 0.009), higher Charlson score (HR 1.52, 95% CI 1.29–1.79; *p* < 0.001), poorly differentiated carcinoma (HR 1.61, 95% CI 1.08–2.39; *p* = 0.02), mucinous carcinoma (HR 3.53, 95% CI 1.72–7.24; *p* < 0.001), and pathological T1 (HR 1.45, 95% CI 1.14–1.84; *p* = 0.002) were independent predictors of increased mortality. LE did not correlate with worse OS (HR 0.91, 95% CI 0.42–1.97; *p* = 0.82).

**Conclusion:**

Our findings show no overall significant survival difference between LE and total mesorectal excision, including ypT1 tumors. Moreover, patients with poorly differentiated or mucinous adenocarcinomas generally had poorer outcomes, regardless of surgical method.

## Introduction

Approximately 150,000 new cases of colorectal cancer (CRC) are diagnosed annually in the USA, with rectal cancer accounting for approximately one-third of cases [1]. During the last few decades, the management of rectal cancer has notably changed [2]. The standardization of surgical treatment of rectal cancer started with the introduction of total mesorectal excision (TME) [3] and the widespread use of neoadjuvant radiotherapy [4], which allowed for better local disease control and a significant reduction in local recurrence following surgical treatment [5].

Radical surgery remains the cornerstone of treatment of rectal cancer [6–11]. However, it may be followed by a significant negative impact on quality of life. Radical surgery has short-term problems, including a morbidity rate of up to 50% and the frequent need for an ileostomy. In addition, long-term significant changes in bowel habits are expected [12–14]. These important issues motivated researchers to seek alternatives to radical surgery in patients with a clinical complete and near-complete response to neoadjuvant therapy [11]. The concept of organ preservation in rectal cancer, first introduced by Professor Habr-Gama and colleagues in 1998 [8], allowed patients to avoid radical surgery and its associated morbidity if they had a clinical complete response and were closely followed up [15, 16]. Furthermore, subsequent studies demonstrated that, in patients with recurrence, salvage proctectomy could be performed with comparable outcomes to patients who underwent proctectomy [17, 18].

During the last decade, researchers have tried to expand the criteria for organ preservation in rectal cancer to include not only patients with a clinical complete response but also patients with a significant downstaging of the tumor. Several randomized trials examined the role of local excision of tumor remnant in patients with T0–T1 tumors, with conflicting results [19–21]. Although the overall survival was comparable to patients who underwent radical surgery and the recurrence rate remained low, patients with recurrence who initially underwent local excision followed by salvage proctectomy surgery had worse postoperative morbidity compared to patients who underwent upfront proctectomy surgery [20]. However, these studies were relatively small and the need for large-scale database analyses encouraged us to investigate this issue using real-world data from one of the largest oncological databases, the National Cancer Database (NCDB), a joint project of the Commission on Cancer (CoC) of the American College of Surgeons and the American Cancer Society.

This study aimed to assess the short- and long-term outcomes of patients with rectal cancer treated with neoadjuvant therapy and local excision compared to proctectomy. We hypothesized that the outcomes available for assessment would be similar; however, given the less invasive nature of local excision, we believe that short-term outcomes following surgery would emphasize the value of this novel therapeutic approach.

## Methods

### Study design

We conducted a retrospective analysis of the NCDB, including all patients with rectal cancer in the database over 16 years (2004–2019). The NCDB is a national database that comprises patients afflicted with different types of cancer, derived from hospital registry data from over 1500 CoC-accredited hospitals across the USA.

### Ethical consideration

Owing to the public nature of the NCDB, which includes de-identified patient data, and the study’s retrospective nature, ethics approval and written consent to participate in the study were not required.

### Data review and selection criteria

Two investigators reviewed the NCDB Participant User File (PUF). We included all patients with rectal cancer with a pathology of adenocarcinoma, mucinous adenocarcinoma, and signet ring cell carcinoma, excluding other histologic types of rectal cancer. For this study, we included all patients with locally advanced disease (clinical TNM stage I–III rectal cancer) treated with neoadjuvant therapy and a final pathology of ypT0–1. We excluded the following patients

- Patients with metastatic disease or patients with an unknown metastatic status
- Patients that did not undergo a surgical intervention or that underwent surgery but without specification of the type of surgical intervention
- Patients who were not treated with neoadjuvant therapy prior to surgery or with an unknown neoadjuvant status
- Patients with a pathological T2–4 or unknown pathological T stage status

Patients were then classified into two groups: patients who underwent local excision as their definitive surgical intervention (LE group) and patients who underwent proctectomy (proctectomy group) as their definitive procedure. After an initial analysis of the unmatched groups, we matched the two groups using the nearest-neighbor propensity score matching method with 1:1 allocation and a caliper of 0.2. The criteria used for the propensity matching included demographic factors such as patient age, sex, Charlson comorbidity index score, race, insurance status, and patient geographic residential area, and clinical factors such as tumor grade, histology type, tumor stage, neoadjuvant and adjuvant therapy.

### Data collected

The following data were collected and used for the analysis:

- Baseline characteristics: age, sex, race, Charlson score, clinical TNM stage
- Insurance status and residence area
- Pathologic TNM stage, tumor histology, grade, lymphovascular invasion, number of lymph nodes examined, and positive lymph nodes in the proctectomy group
- Treatment details: chemotherapy, radiotherapy, sequencing of systemic and radiation therapy, type of surgery, and days from diagnosis to surgery
- Outcomes: the primary outcome was overall survival. Secondary outcomes included 30- and 90-day mortality, 30-day readmission, and follow-up duration

### Statistical analysis

Statistical analyses were performed using EZR (version 1.55) and R software (version 4.1.2). [22] Continuous data were expressed as mean and standard deviation when normally distributed or otherwise as the median and interquartile range (IQR). Student *t* test or Mann–Whitney *U* test was used to analyze continuous variables. Categorical data were expressed as numbers and proportions and analyzed using Fisher exact or chi-square test. Kaplan–Meier statistics and log-rank tests were used to detect differences in overall survival between the groups. A *p* value < 0.05 was considered significant. The CoC’s NCDB and the participating hospitals were the sources of the de-identified data used herein; they have not verified and are not responsible for the statistical validity of the data analysis or the conclusions derived by the authors.

## Results

**Fig. 1:**
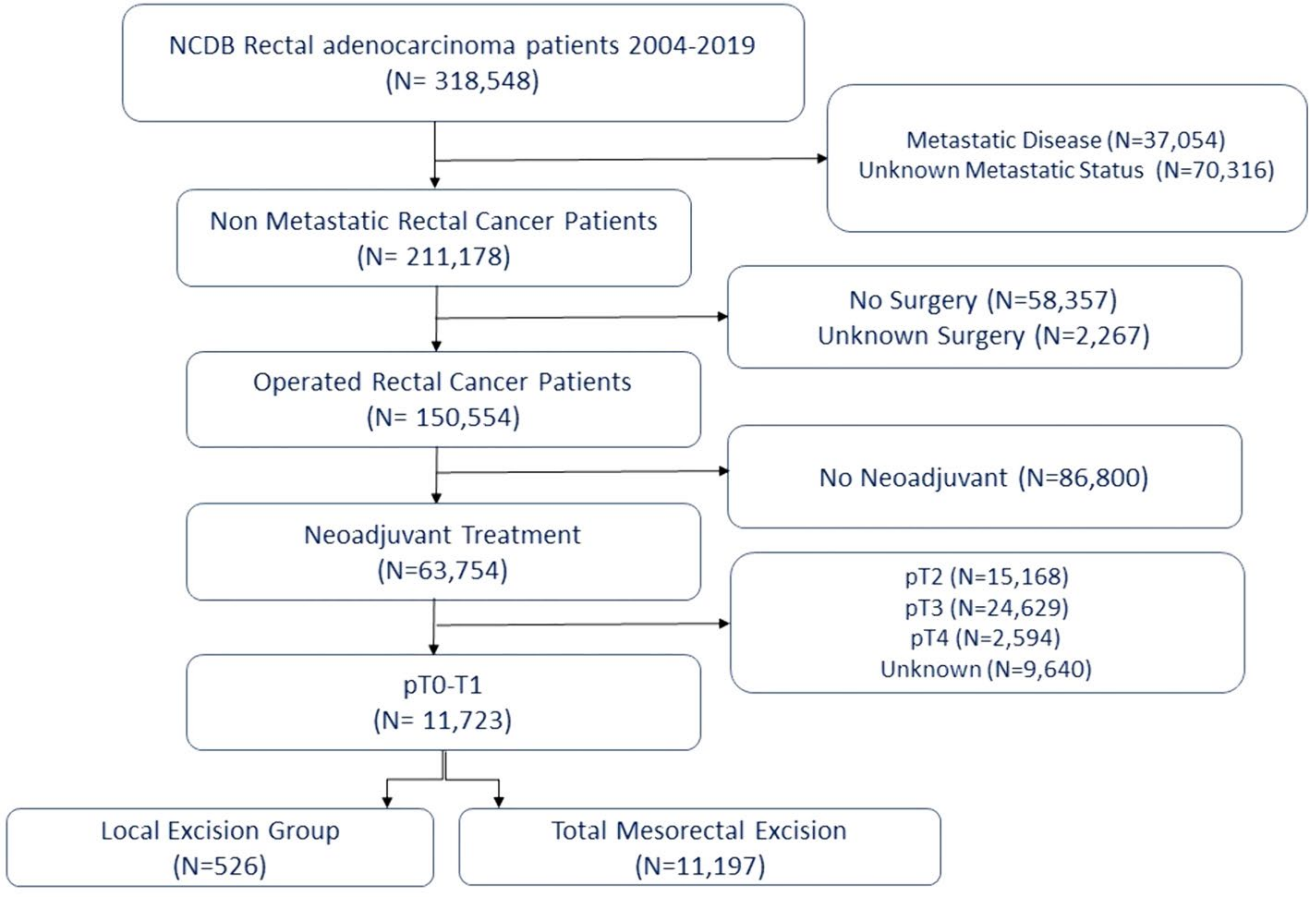
Study flowchart of included patients

Overall, 11,256 out of 318,548 patients (3.5%) with non-metastatic pathological T0–T1 rectal adenocarcinomas treated with neoadjuvant therapy from the NCDB database were included. Of these patients, 526 (4.6%) underwent local excision as their definitive surgery, while 10,730 (95.4%) patients underwent proctectomy (Fig. 1).

### Patient characteristics: before matching

Analysis of the original unmatched groups demonstrated that patients who underwent local excision were significantly older (66 vs. 60 years, *p* < 0.001) and were less likely to have private insurance (*p* < 0.001), but comparable in sex (*p* = 0.71), race (*p* = 0.26), Charlson index score (*p* = 0.17), and residence area (*p* = 0.4). As for clinical presentation, patients in the proctectomy group presented with a more advanced clinical staging, including more T3 and T4 tumors (*p* < 0.001) and more N1–2 staging (*p* < 0.001). Furthermore, patients in the proctectomy group received more chemotherapy compared to the LE group (95.3% vs. 88.8%; *p* < 0.001). Among patients who underwent proctectomy and had an ypT0 (*n* = 7361), 589 patients (8%) had a positive ypN (N1 in 519 patients and N2 in 70 patients). Among patients with ypT1 (*n* = 3843), 562 patients (14.6%) had positive nodal disease (N1 in 489 patients and N2 in 73 patients). A summary of the characteristics of unmatched patients in the LE and proctectomy groups is shown in Table 1.

**Table 1:** Unmatched comparison of demographic and clinical characteristics between patients with rectal cancer that underwent local excision compared to proctectomy surgery

Factor	Local excision group (*n* = 526)	Proctectomy group (*n* = 10,730)	*P* value
Age, years, median (range)	66 (35–90)	60 (19–90)	< 0.001
Sex (%)			0.715
Female	202 (38.4)	4210 (39.2)	
Male	324 (61.6)	6520 (60.8)	
Race (%)			0.26
White	463 (88.2)	9378 (87.9)	
Black	35 (6.7)	725 (6.8)	
Asian	16 (3.0)	411 (3.9)	
Other	6 (1.2)	118 (1.1)	
Residence area			0.4
Metro	399 (78.9)	8305 (81.2)	
Rural	13 (2.6)	248 (2.4)	
Urban	94 (18.6)	1681 (16.4)	
Charlson score (%)			0.176
0	392 (74.5)	8342 (77.7)	
1	94 (17.9)	1782 (16.6)	
2	29 (5.5)	419 (3.9)	
3	11 (2.1)	187 (1.7)	
Type of insurance (%)			< 0.001
Medicaid	22 (4.2)	651 (6.1)	
Medicare	256 (49.3)	3843 (36.3)	
Private	229 (44.1)	5693 (53.7)	
Other	4 (0.8)	114 (1.1)	
Not insured	8 (1.5)	293 (2.8)	
Grade (%)			0.419
Well differentiated	49 (12.3)	837 (10.1)	
Moderately differentiated	311 (77.9)	6721 (80.8)	
Poorly differentiated	36 (9.0)	705 (8.5)	
Undifferentiated	3 (0.8)	51 (0.6)	
Histology (%)			0.877
Adenocarcinoma	516 (98.1)	10,486 (97.7)	
Mucinous adenocarcinoma	7 (1.3)	181 (1.7)	
Signet ring cell adenocarcinoma	6 (0.6)	63 (0.6)	
Median tumor size (mm)	30 (20–40)	40 (27–50)	< 0.001
Lymphovascular invasion (%)	9 (4.2)	223 (4.2)	1
Clinical T stage (%)			< 0.001
1	71 (13.7)	602 (5.7)	
2	137 (26.5)	1287 (12.1)	
3	258 (49.9)	7703 (72.4)	
4	5 (1.0)	388 (3.6)	
Clinical N stage (%)			< 0.001
0	371 (71.3)	5295 (49.7)	
1	113 (21.7)	4240 (39.8)	
2	14 (2.7)	779 (7.3)	
TNM stage group (%)			< 0.001
0	4 (0.8)	50 (0.5)	
1	179 (37.2)	961 (9.6)	
2	171 (35.6)	4015 (40.2)	
3	127 (26.4)	4955 (49.6)	
Pathological N status (%)			NA
ypT0N1	NA	519 (7)	
ypT0N2	NA	70 (0.9)	
ypT1N1	NA	489 (12.7)	
ypT1N2	NA	73 (1.9)	
Chemotherapy (%)	467 (88.8)	10,224 (95.3)	< 0.001
Duration of radiation, days, median	40	39	0.02
Days from diagnosis to definitive surgery, median	147	137	< 0.001
Type of surgery (%)			< 0.001
Local excision	526 (100)		
Abdominoperineal resection		2531 (23.6)	
Anterior resection		7110 (66.3)	
Proctectomy with pull through coloanal anastomosis		83 (0.8)	
Restorative proctectomy		1006 (9.4)	
Follow-up, months, mean (SD)	75.9 (42.77)	75.2 (50.62)	0.67

*SD* standard deviation; *NA* not available

### Propensity score matched analysis

After propensity score matching, patients in the two groups had similar demographic, clinical, and therapeutic factors, as shown in Table 2.

**Table 2:** Propensity score matched analysis of patients with rectal cancer treated with neoadjuvant therapy and underwent local excision compared to proctectomy surgery

Factor	Matched local excision group (*n* = 342)	Matched proctectomy group (*n* = 342)	*P* value
Age, years, median (range)	65 (36–90)	65 (40–90)	0.82
Sex (%)			0.69
Female	132 (38.6)	126 (36.8)	
Male	210 (61.4)	216 (63.2)	
Race (%)			0.55
White	308 (90.1)	296 (86.5)	
Black	17 (5.0)	27 (7.9)	
Asian	12 (3.5)	13 (3.8)	
Other	5 (1.5)	6(1.8)	
Geographic classification			0.80
Metro	271 (79.2)	263 (76.9)	
Rural	7 (2.0)	8 (2.3)	
Urban	64 (18.7)	71 (20.8)	
Charlson score (%)			0.36
0	262 (76.6)	265 (77.5)	
1	58 (17.0)	60 (17.5)	
2	20 (5.8)	12 (3.5)	
3	2 (0.6)	5 (1.5)	
Type of insurance (%)			0.70
Medicaid	12 (3.5)	17 (5.0)	
Medicare	163 (47.7)	156 (45.6)	
Private	159 (46.5)	157 (45.9)	
Other	3 (0.9)	3 (0.9)	
Not insured	5 (1.5)	9 (2.6)	
Grade (%)			0.56
Well differentiated	40 (11.7)	49 (14.3)	
Moderately differentiated	266 (77.8)	262 (76.6)	
Poorly differentiated	33 (9.6)	30 (8.8)	
Undifferentiated	3 (0.9)	1 (0.3)	
Histology (%)			1
Adenocarcinoma	334 (97.7)	334 (97.7)	
Mucinous adenocarcinoma	6 (1.8)	7 (2.0)	
Signet ring cell adenocarcinoma	2 (0.6)	1 (0.3)	
Tumor size, mm, median (range)	30 (20–40)	36 (28–50)	0.003
Lymphovascular invasion (%)	8 (5.7)	9 (5.1)	1
Clinical T stage (%)			0.054
1	50 (14.7)	62 (18.1)	
2	102 (29.9)	89 (26.0)	
3	179 (52.5)	174 (50.9)	
4	3 (0.9)	12 (3.5)	
Clinical N stage (%)			0.602
0	248 (72.5)	249 (72.8)	
1	82 (24.0)	75 (21.9)	
2	8 (2.3)	14 (4.1)	
TNM stage group (%)			0.647
0	2 (0.6)	5 (1.5)	
1	132 (38.6)	137 (40.1)	
2	118 (34.5)	109 (31.9)	
3	90 (26.3)	91 (26.6)	
4	0 (0.0)	0 (0.0)	
Chemotherapy (%)	314 (91.8)	326 (95.3)	0.08
Duration of radiation, days, median	40	39	0.17
Days from diagnosis to definitive surgery, median	146	136	0.002
Type of surgery (%)			< 0.001
Local excision	342 (100)	78 (22.8)	
Abdominoperineal resection		233 (68.1)	
Anterior resection		28 (8.2)	
Proctectomy with pull through coloanal anastomosis		3 (0.9)	
Restorative proctectomy		78 (22.8)	
Follow-up, months, median	68.7	67.1	0.20

**Fig. 2:**
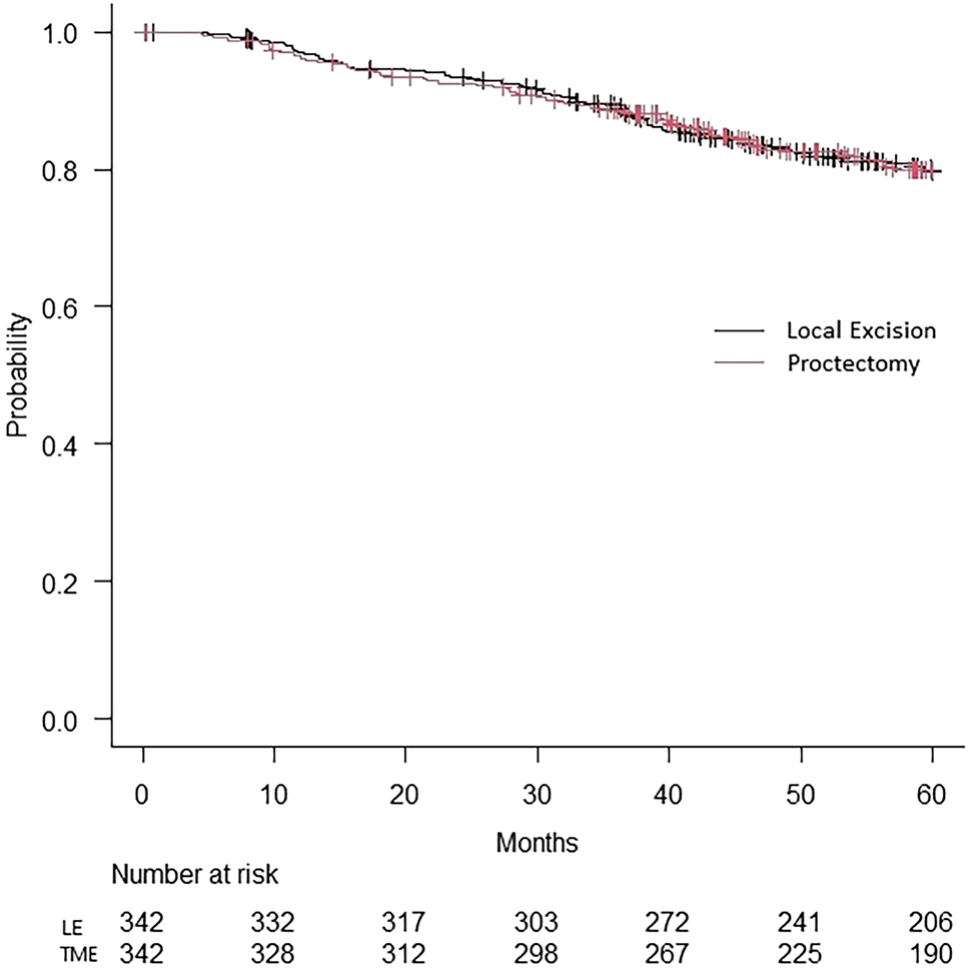
Kaplan–Meier survival curve of propensity score matched patients that underwent local excision as their definitive surgery compared to patients that underwent TME surgery

According to the Kaplan–Meier survival with log rank test, the overall survival was similar between the groups, with a mean of 54.1 months in the LE group compared to 54.2 months in the proctectomy group (*p* = 0.881) (Fig. 2).

Analysis of secondary outcomes demonstrated that positive resection margins (1.2% vs. 0.6%;* p* = 0.45), pathologic T stage (*p* = 0.07), 30-day mortality (0.6% vs. 0.6%; *p* = 1), and 90-day mortality (1.5% vs. 1.2%; *p* = 0.75) were comparable between the groups. The median length of stay (1 vs. 6 days; *p* < 0.001) was shorter and 30-day readmission rate (5.3% vs. 10.3%; *p* = 0.02) was lower in the LE group, compared to proctectomy (Table 3).

**Table 3:** Propensity score matched analysis of patient outcomes

Factor	Matched local excision group (*n* = 342)	Matched proctectomy group (*n* = 342)	*P* value
Surgical margins (%)			0.45
Negative	335 (98.8)	339 (99.4)	
Positive	4 (1.2)	2 (0.6)	
Pathological T stage (%)			0.07
0	171 (50)	199 (58.2)	
In situ	17 (5)	18 (5.3)	
1	154 (45)	125 (36.5)	
Length of stay, days, median (range)	1 (0–115)	6 (3–51)	< 0.001
30-day readmission (%)	18 (5.3)	34 (10.3)	0.02
30-day mortality (%)	2 (0.6)	2 (0.6)	1
90-day mortality (%)	5 (1.5)	4 (1.2)	0.75
Overall survival (%)	229 (67)	248 (72.5)	0.13

*TME* total mesorectal excision

### Sub-analysis of the ypT1 group

**Fig. 3:**
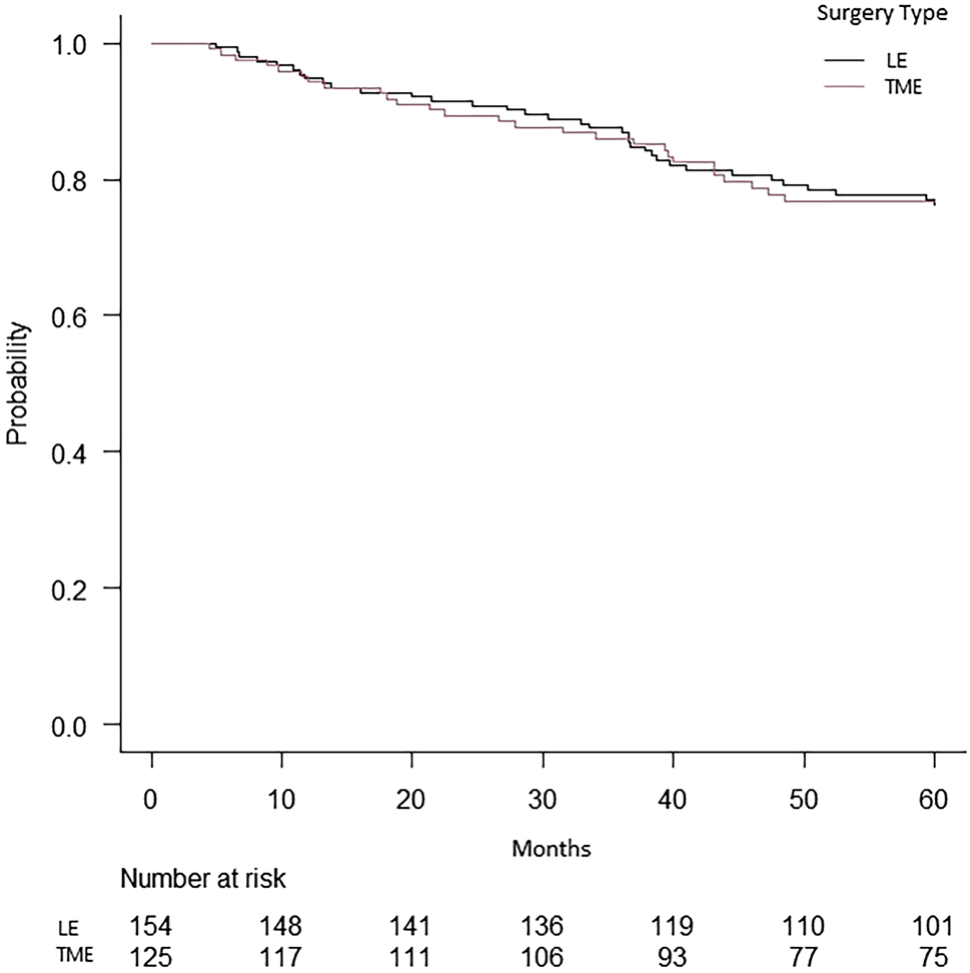
Kaplan–Meier survival curve of propensity score matched patients with ypT1 pathology that underwent local excision as their definitive surgery compared to patients that underwent TME surgery. LE, local excision; TME, total mesorectal excision

Within the matched cohort, 279 patients had pT1 lesions, with the majority treated by local excision (*n* = 154, 55.2%). Survival analysis showed no differences in the mean 5-year overall survival (52.9 months for local excision vs. 52.4 months for proctectomy; *p* = 0.78) (Fig. 3). Furthermore, no differences were seen in the positive margin rates or 30- and 90-day mortality rates (*p* = 1). The only difference between the groups was in the median length of stay following surgery (1 day for local excision vs. 5 days for proctectomy, *p* < 0.001). Analysis of secondary outcomes for the ypT1 group can be seen in Table 4.

**Table 4:** Propensity score matched sub-analysis of patients with ypT1 pathology

Factor	Matched local excision group (*n* = 154)	Matched proctectomy group (*n* = 125)	*P* value
Surgical margins (%)			1
Negative	152 (98.7)	123 (98.4)	
Positive	2 (1.3)	2 (1.6)	
Length of stay, days, median (range)	1 (0–94)	5 (0–26)	< 0.001
30-day readmission (%)	7 (4.5)	4 (3.2)	0.127
30-day mortality (%)	2 (1.3)	1 (0.8)	1
90-day mortality (%)	4 (2.6)	3 (2.4)	1

### Factors associated with overall survival

In the univariate Cox proportional regression analysis (Table 5), several factors were associated with lower survival, including age (HR 1.061, 95% CI 1.057–1.065; *p* < 0.001), male sex (HR 1.37, 95% CI 1.265–1.503, *p* < 0.001), Charlson score (HR 1.61, 95% CI 1.535–1.698; *p* < 0.001), poorly differentiated histology (HR 1.53, 95% CI 1.32–1.76; *p* < 0.001), and positive nodal status (HR 1.64, 95% CI 1.41–1.91; *p* < 0.001), while others, including chemotherapy (HR 0.77, 95% CI 0.7306–2.271; *p* < 0.001) and local excision (HR 0.63, 95% CI 0.5451–0.7448; *p* < 0.001), were correlated with increased survival.

**Table 5:** Cox regression univariate analysis of factors correlated with overall survival

Factor	Group	Alive (*n* = 8912)	Dead (*n* = 2344)	HR	95% CI	*P* value
Age, years, median (range)		59 (19–90)	68 (20–90)	1.061	1.057–1.065	< 0.001*
Sex (%)	Female	3642 (40.9)	770 (32.8)	Ref	–	–
	Male	5270 (59.1)	1574 (67.2)	1.37	1.265–1.503	< 0.001*
Race (%)	White	7761 (87.6)	2080 (89.0)	1.079	0.5799–2.009	0.809
	Black	585 (6.6)	175 (7.5)	1.174	0.6207–2.220	0.621
	Asian	367 (4.1)	60 (2.6)	0.751	0.3849–1.469	0.809
	Other	112 (1.3)	12 (0.5)	Ref	–	–
Charlson Deyo score (%)	0	7194 (80.7)	1540 (65.7)	1.614	1.535–1.698	< 0.001*
	1	1333 (15.0)	543 (23.2)			
	2	273 (3.1)	175 (7.5)			
	3	112 (1.3)	86 (3.7)			
Geographic region (%)	Metropolitan	6957 (82.1)	1747 (77.2)	Ref	–	–
	Urban	215 (2.5)	46 (2.0)	1.4420	1.3030–1.597	< 0.001*
	Rural	1305 (15.4)	470 (20.8)	0.8638	0.6446–1.158	< 0.001*
Insurance status (%)	Medicaid	566 (6.4)	107 (4.6)	Ref	–	–
	Medicare	2714 (30.8)	1385 (59.9)	2.0540	1.6870–2.5000	< 0.001*
	Other government	94 (1.1)	24 (1.0)	1.1450	0.7353–1.7820	< 0.001*
	Private	5196 (59.0)	726 (31.4)	0.6405	0.5228–0.7847	< 0.001*
	Not insured	230 (2.6)	71 (3.1)	1.3320	0.9867–1.7980	< 0.001*
Clinical TNM (%)	0	37 (0.4)	17 (0.8)	Ref	–	–
	1	826 (9.9)	314 (14.9)	0.8838	0.5424–1.440	0.619
	2	3272 (39.1)	914 (43.4)	0.8003	0.4952–1.294	0.363
	3	4225 (50.5)	857 (40.7)	0.6695	0.4141–1.082	0.101
Pathological T (%)	0	5977 (50.9)	1385 (11.8)	Ref	–	–
	IS	400 (3.4)	120 (1)	1.290	1.040–1.610	0.02*
	1	2897 (24.7)	945 (8)	1.430	1.300–1.570	< 0.001*
Pathological N (%)	N0	8056 (68.7)	1981 (16.9)	Ref	–	–
	N+	1177 (7.3)	312 (2.6)	1.640	1.410–1.910	< 0.001*
Grade (%)	Well differentiated	683 (9.9)	203 (11.2)	1.1910	1.0280–1.380	0.02*
	Moderately differentiated	5640 (81.8)	1392 (76.6)	Ref	–	0.245
	Poorly differentiated	524 (7.6)	217 (11.9)	1.5310	1.3270–1.766	< 0.001*
	Undifferentiated	48 (0.7)	6 (0.3)	0.5572	0.2499–1.242	0.1529
Histology (%)	Adenocarcinoma	8758 (98.3)	2244 (95.7)	Ref	–	–
	Mucinous carcinoma	116 (1.3)	72 (3.1)	1.984	1.569–2.509	< 0.001*
	Signet ring cell carcinoma	38 (0.4)	28 (1.2)	2.514	1.731–3.650	< 0.001*
Lymphovascular invasion (%)	No	4378 (96.1)	871 (94.3)	Ref	–	–
	Yes	179 (3.9)	53 (5.7)	1.485	1.125–1.96	0.005*
Tumor size, mm, median (range)		40 (20–50)	40 (20–58)	1	1–1.001	0.04*
Chemotherapy (%)	Not given	356 (4.0)	208 (8.9)	Ref	–	–
	Given	8555 (96.0)	2136 (91.1)	0.774	0.7306—2.271	< 0.001*
Type of surgery (%)	Local excision	356 (4.0)	170 (7.3)	0.6372	0.5451–0.7448	< 0.001*
	TME	8556 (96.0)	2174 (92.7)	Ref	–	–
Surgical margins (%)	Negative	8796 (99.2)	2285 (98.2)	Ref	–	–
	Positive	69 (0.8)	43 (1.8)	1.867	1.38–2.52	< 0.001*

*HR* hazard ratio, *CI* confidence interval

*P value indicates statistical significance

Multivariate analysis of statistically significant factors in the univariate analysis (Table 6) demonstrated that male sex (HR 1.38, 95% CI 1.08–1.77; *p* = 0.009), higher Charlson score (HR 1.52, 95% CI 1.29–1.79; *p* < 0.001), poorly differentiated carcinoma (HR 1.61, 95% CI 1.08–2.39; *p* = 0.02), mucinous carcinoma (HR 3.53, 95% CI 1.72–7.24; *p* < 0.001), pathological T1 (HR 1.45, 95% CI 1.14–1.84; *p* = 0.002), and pathological positive nodes (HR 1.94, 95% CI 1.28–2.93; *p* = 0.001) were among the independent predictors of increased mortality. Chemotherapy (HR 0.82, 95% CI 0.41–1.62; *p* = 0.56) and local excision (HR 0.91, 95% CI 0.42–1.97; *p* = 0.82) were not found to be significant predictors of survival.

**Table 6:** Multivariate Cox regression analysis of factors correlated with overall survival

Variable	HR	95% CI	*P* value
Age	1.06	1.05–1.08	< 0.001*
Sex	1.38	1.08–1.77	0.009*
Charlson score	1.52	1.29–1.79	< 0.001*
Geographic region			
Urban	1.51	1.13–2.02	0.005*
Rural	0.49	0.18–1.3	0.15
Insurance status			
Medicare	0.61	0.36–1.03	0.06
Private	0.59	0.37–0.94	0.02*
Other	0.34	0.09–1.28	0.11
No insurance	0.38	0.13–1.13	0.07
Grade			
Well differentiated	1.04	0.71–1.5	0.85
Poorly differentiated	1.61	1.08–2.39	0.02*
Undifferentiated	0.000	0–Inf	0.97
Histology			
Mucinous	3.53	1.72–7.24	< 0.001*
Signet ring cell	1.09	0.17–6.89	0.92
Lymphovascular Invasion	1.25	0.7–2.2	0.44
Chemotherapy	0.82	0.41–1.62	0.56
Tumor size	1	0.999–1.001	0.02*
Local excision	0.91	0.42–1.97	0.82
Positive surgical margins	0.92	0.29–2.86	0.89
Pathological T			
In situ	1.05	0.6–1.84	0.85
1	1.45	1.14–1.84	0.002*
Pathological N+	1.94	1.28–2.93	0.001*

*HR* hazard ratio, *CI* confidence interval

*P value indicates statistical significance

## Discussion

In this study, we aimed to analyze the short- and long-term outcomes of patients with T0–T1 rectal adenocarcinoma who were treated with neoadjuvant chemoradiotherapy. After propensity score matching for various demographic, clinical, and therapeutic parameters, we found that patients who underwent local excision of a T0–T1 tumor had similar survival to proctectomy surgery. Furthermore, multivariate analysis did not identify local excision as a predictor of reduced or improved overall survival. Conversely, male patients with higher Charlson score and poorly differentiated and/or mucinous adenocarcinomas, and patients with pathological positive nodes who underwent proctectomy may have significantly reduced overall survival.

Organ preservation is a relatively new concept in rectal cancer management. It was first introduced for patients with a clinical complete response after neoadjuvant radiotherapy. This strategy includes the preservation of the rectum with close clinical and endoscopic follow-up for early detection of possible tumor recurrence [23]. In addition, in some patients, local excision of a remnant scar is performed to ensure the complete pathological response following treatment [24, 25]. Local excision may be considered a safe alternative to radical resection in patients with complete tumor response; therefore, it is incorporated into the major treatment guidelines, including the National Accreditation Program for Rectal Cancer (NAPRC) and the National Comprehensive Cancer Network (NCCN) recommendations [26, 27]. However, there is an ongoing debate about the safety of this therapeutic strategy in pTis-T1 tumors because no lymph nodes are examined, and thus the nodal status remains unknown [28].

Several retrospective and prospective studies assessed the outcomes of local excision of rectal cancers after response to neoadjuvant therapy. A pooled analysis of 20 studies, including more than 1000 patients who underwent local excision following neoadjuvant therapy [11], found that the overall disease-free survival of patients with pT1 tumors was 68%, with a recurrence rate of 21%. However, this pooled analysis combined data from multiple small-scale studies with significant heterogeneity. In addition, Garcia-Aguilar et al. [19] conducted a multicenter randomized control phase 2 trial (ACOSOG Z6041). Patients with T2N0 distal rectal cancer tumors underwent local excision following neoadjuvant therapy, with a disease-free survival rate of 88.2% in the intention-to-treat group. These studies, among others, also pointed out that the most common salvage proctectomy for recurrence after local excision was abdominoperineal resection, which adds to the complex array of factors engulfed in the therapeutic decision-making process. [29]

Another important phase 3 randomized multicenter controlled trial (GRECCAR 2) from France [20] included patients with stage T2–T3 lower rectal carcinoma, who had a good clinical response to neoadjuvant chemoradiotherapy. Patients were randomized to undergo local excision or proctectomy, and patients with a final pathology of T2–T3 in the local excision group underwent completion proctectomy. The authors found no differences in oncological and functional outcomes between the two groups; however, 35% of patients who underwent LE required proctectomy completion. Furthermore, the study demonstrated that patients in need of completion proctectomy are prone to significant morbidity following surgery. A follow-up study examined the oncological outcomes and demonstrated no differences in 5-year overall survival for the same cohort [21].

Our study highlights some important advantages local excision may have over radical surgery, especially in patients with ypT0. We found that the length of stay was significantly shorter in the LE group (1 vs. 6 days, *p* < 0.001), which is not surprising given the high morbidity that proctectomy carries. In addition, we can assume that for a large portion of the proctectomy group, a protective ostomy was performed to allow anastomotic healing [30]. Furthermore, we noticed that the readmission rate was almost half in the LE group compared to the proctectomy group (5.3% vs. 10.3%; *p* = 0.02). Although the NCDB data does not detail postoperative complications, we can assume that the reason for the higher rate of readmissions is due to the complexity of proctectomy and probably also due to ostomy issues, which are common causes for readmission following these procedures [31, 32], not to mention the need for an additional surgical intervention to restore bowel continuity. We must also consider the significant changes in bowel movements and the associated impact on patients’ quality of life, which are very common following restorative proctectomy and are far less common following local excision [33]. Given that the survival outcomes are similar between the two groups in our analysis and the previously mentioned studies, the advantages of local excision and organ preservation should be taken into account by caregivers and patients alike; however, caution must be taken in patients with residual T1 disease seen on pathology. These patients should be considered for salvage TME to increase the likelihood of survival.

Our study has several limitations, most of which stem from the nature of the NCDB data. Unfortunately, the NCDB does not offer progression-free survival and disease recurrence data, precluding our ability to report these important outcomes. The retrospective nature of the NCDB precludes the ability to examine in real-time the decision-making process and how local excision was chosen over proctectomy, or if local excision was attempted first and was then followed by proctectomy if needed, as the NCDB records include the definitive surgery alone. Furthermore, no data was available on the functional outcomes or the quality of life of patients.

This large-scale database and its longevity give us a glimpse of the current treatment of rectal cancer, demonstrating that local excision was not commonly used in this setting, as only 4% of patients underwent local excision. Furthermore, the large-scale database enabled a robust propensity matching, which confirms that organ preservation is feasible and has similar survival outcomes in one of the largest comparative cohorts reported to date. It strengthens the conclusion of previously randomized published trials that organ preservation is feasible and is not associated with worse oncological outcomes.

## Conclusion

Our findings show no overall significant survival difference between LE and total mesorectal excision for all patients, including patients with ypT1 tumors. Moreover, patients with poorly differentiated or mucinous adenocarcinomas generally had poorer outcomes, regardless of the surgical method employed.

## Data Availability

Upon reasonable request to first author.
